# “Revisiting the past”: a redescription of *Physaloptera retusa* (Nemata, Physalopteridae) from material deposited in museums and new material from Amazon lizards

**DOI:** 10.1590/S1984-29612023016

**Published:** 2023-03-27

**Authors:** Lílian Cristina Macedo, Yuri Willkens, Leandro Maurício Oliveira Silva, Scott Lyell Gardner, Francisco Tiago de Vasconcelos Melo, Jeannie Nascimento dos Santos

**Affiliations:** 1 Laboratório de Ecologia e Conservação da Amazônia, Universidade Federal Rural da Amazônia - UFRA, Capitão Poço, PA, Brasil; 2 Laboratório de Biologia Celular e Helmintologia “Profa. Dra. Reinalda Marisa Lanfredi”, Instituto de Ciências Biológicas, Universidade Federal do Pará - UFPA, Belém, PA, Brasil; 3 Harold W. Manter Laboratory of Parasitology, University of Nebraska, Lincoln, Nebraska, United States

**Keywords:** Helminths of reptiles, Amazon, taxonomical identification, morphology, scanning electron microscopy, Helmintos de répteis, Amazônia, identificação taxonômica, morfologia, microscopia eletrônica de varredura

## Abstract

*Physaloptera* Rudolphi, 1819 is a genus of nematodes that includes approximately 100 species parasitic in vertebrates around the world. From these, approximately 30 occur in the Neotropical region, with nine reported from neotropical reptiles. *Physaloptera* spp. are recognized by their distinct morphology of the apical end and characters of the reproductive system. However, despite the fact that the morphological characters for species diagnosis have been firmly established, we frequently find identification problems regarding poorly detailed descriptions and poorly preserved specimens. These may lead to taxonomic incongruencies. *Physaloptera retusa* (Rudolphi, 1819) is the most common species of the genus and has been reported from several species of neotropical reptiles. Based on our reexaminations of nematode specimens identified as *P. retusa* from different museum collections, we provide a detailed redescription including the type material, voucher specimens and new specimens recovered currently and showed in this study with new morphological data obtained using light and scanning electron microscopy tools.

## Resumo

*Physaloptera* Rudolphi, 1819 é um gênero de nematódeos que inclui aproximadamente 100 espécies parasitárias em vertebrados em todo o mundo. Destes, aproximadamente 30 ocorrem na região Neotropical, e nove foram reportados para répteis neotropicais. *Physaloptera* spp. são reconhecidas por sua morfologia distinta na extremidade apical e por caracteres do sistema reprodutivo, especialmente nos machos. No entanto, embora os caracteres morfológicos para o diagnóstico de espécies tenham sido estabelecidos, frequentemente são encontrados problemas de identificação em relação a descrições pouco detalhadas e espécimes mal preservados. Isto pode levar a incongruências taxonômicas e erros de identificação. *Physaloptera retusa* (Rudolphi, 1819) é a espécie mais comum do gênero e tem sido reportada para várias espécies de répteis neotropicais. Com base nos estudos das revisões de espécimes de nematódeos, identificados como *P. retusa* de diferentes coleções de museus, foi providenciada uma redescrição detalhada, incluindo-se o material-tipo, espécimes "voucher" e novos espécimes obtidos neste estudo com novos dados morfológicos, obtidos a partir de microscopia eletrônica de luz e varredura.

## Introduction

The genus *Physaloptera* Rudolphi, 1819 (Nemata: Physalopteridae) includes approximately 100 species of nematode parasites of vertebrates distributed worldwide (Pereira et al., 2014). Of those, the Neotropics have 30 species of *Physaloptera* (Pereira et al., 2012), and nine of these parasitize reptiles (Pereira et al., 2014; São Luiz et al., 2015; Matias et al., 2020).

*Physaloptera* spp. identification is based mainly on the number and disposition of teeth, the relative position of the excretory pore and deirids, the relative position of the vulva in females, the pattern and number of caudal papillae and the shape and size of the spicules in males (Ortlepp, 1922; Morgan, 1943; Skrjabin & Sobolev, 1964; Esteban et al., 1995; Lopes-Torres et al., 2009). However, many species in this genus were inaccurately or superficially described during the taxonomic history of the genus. The differential diagnosis of species was based on different morphological characters in the past years, which caused difficulties to establish which are taxonomically informative (Alves et al., 2022). Furthermore, problems regarding insufficiently detailed descriptions (especially in species described more than a century ago, limited by the technology of the time) and studies based on poorly preserved specimens, presenting technical artifacts, may lead to inaccurate identifications (Pereira et al., 2012, 2014; Davis et al., 2016).

*Physaloptera retusa* (Rudolphi, 1819) was described in *Tupinambis teguixin* (Linnaeus, 1758) (Squamata, Teiidae) from Brazil. Then, Ortlepp (1922) redescribed *P. retusa* including material obtained from a specimen of *T. teguixin* found dead in the Gardens of the London Zoological Society. Alves et al. (2022) recently studied this species using material collected in the State of Minas Gerais, Brazil, and voucher specimens from the Helminthological Collection of the Oswaldo Cruz Institute. However, there is no recent studies regarding the morphology of type specimens.

We present a morphological redescription of *P. retusa*, indicating complementary characters for species differentiation, based on observations of the type series, and specimens identified as *P. retusa* deposited in different museum collections, and from new material obtained in this study using both light and scanning electron microscopy.

## Materials and Methods

We collected 28 nematodes from 15 specimens of *Ameiva ameiva* Linnaeus, 1758 (commonly known as Giant Ameiva or Amazon Racerunner) from the “Osvaldo Rodrigues da Cunha” herpetological collection of Emílio Goeldi Museum (MPEG) collected from Caxiuanã National Forest (Flona Caxiuanã), Melgaço Municipality, Pará, Brazil. The hosts were previously fixed in 3% formaldehyde and stored in 70% ethanol and were dissected at the Laboratory of Cell Biology and Helminthology from the Federal University of Pará (LBCH/ICB/UFPA). The nematodes collected were stored in A.F.A solution (2% acetic acid, 3% formaldehyde and 95% alcohol 70%) and transferred to alcohol 70% after 24 hours.

The nematodes were cleared in lactophenol following the protocols of Gardner et al. (2012) and mounted on temporary slides for morphological analysis using light microscopy. We obtained morphological measurements by drawings using microscopes equipped with camera lucida. All measurements are presented in Table 1 as ranges followed by mean values in parentheses and given in micrometers unless otherwise indicated.

**Table 1:** Comparison of morphometric characters of *Physaloptera retusa*. All measurements are presented as ranges followed by mean values in parentheses when available. Values are given in micrometers unless otherwise stated.

**Reference**	**Rudolphi (1819)** **(Reexamined)**	**Ortlepp (1922)** **(Reexamined)**	**Alves et al. (2022)**	**This study**
**Host**	***Tupinambis teguixin***	***Tupinambis teguixin***	***Tropidurus torquatus***	***Ameiva ameiva***
**Locality**	**Cuiabá (Brazil)**	**Cuiabá (Brazil)**	**Minas Gerais (Brazil)**	**Flona Caxiuanã (Brazil)**
**Males**	(n=5)	(n=4)	(n=5)	(n=7)
Body length (mm)	18-38 (25)	16-22 (18)	13.5-16.1	11-18 (15)
Maximum width	650-800 (720)	650-800 (650)	377-650	343-786 (514)
Esophagus length (mm)	3.6-5.9 (4.7)	3.3-4.4 (3.7)	2.6-2.9	2-5 (4)
Esophagus-body length ratio (%)	16%	20%	16.8-20.2%	13%-28% (23%)
Muscular portion length	490-650 (565)	320-430 (565)	261-450	329-573 (456)
Glandular portion length (mm)	3.1-5.2 (4.2)	3.0-4.0 (3.3)	2.3-2.4	1.9-4.6 (3.150
Nerve ring	200-380 (285)	200-250 (220)	208-380	276-453 (363)
Deirids	380-610 (470)	280-400 (330)	375-435	106-714 (450)
Excretory pore	310-640 (485)	310- 400 (340)	411-500	301-736 (561)
Right spicule	420-470 (450)	230-280 (230)	277-344	231-443 (347)
Left spicule	410-490 (450)	265-310 (265)	287-380	209-667 (406)
Caudal papillae	21 (8 pedunculate, 12 sessile, 1 unpaired)	21 (8 pedunculate, 12 sessile, 1 unpaired)	21 (8 pedunculate, 12 sessile, 1 unpaired)	21 (8 pedunculate, 12 sessile, 1 unpaired)
Tail length/ cloaca to posterior extremity	1.2-1.4 (1.3)	0.9-1.2 (0.9)	547-800	571-974 (788)
Phasmidial pores	490-700 (580)	490-700 (580)	*	*
**Females**	(n=5)	(n=6)	(n=12)	(n=21)
Body length (mm)	20-34 (26)	23-25 (23)	16.8-24.2	17-28 (22)
Maximum width	600-800 (700)	860-980 (900)	498-578	357-853 (645)
Esophagus length	3.5-4.8 (4.1)	3.5-4.8 (3.7)	2.7-3.9	4-6 (5)
Esophagus-body length ratio (%)	14-17 (15)	17	14.4-21	16-31 (21)
Muscular portion length	430-500 (460)	390-610 (390)	275-391	407-616 (510)
Glandular portion length	3.1-4.3 (3.7)	3.1-4.1 (3.3)	2.4-3.6	1520-4933 (3978)
Nerve ring	220-290 (250)	200-220 (220)	282-346	279-533 (417)
Deirids	330-480 (400)	440-520 (520)	410-437	514-906 (610)
Excretory pore	450-490 (465)	380-550 (550)	440-601	547-800 (698)
Vulva opening from anterior extremity	3.2-5.5 (4.3)	4.1-5.5 (5.3)	4.88-7.86	4073-7607 (5778)
Vulva-body length ratio (%)	16%	23%	25.9-36.2	0.17-0.35 (0.27)
Ovijector	1.4-1.8 (1.3)	0.62-0.9 (0.62)		-
Eggs	40-45 × 20-25	43-46 × 20-24	44-46 × 18-21	14-31 (26) × 21-46 (41)
Tail length/ cloaca to posterior extremity	360-440 (395)	275-450 (280)	307 - 532	240-651 (485)
Phasmidial pores	140-170 (150)	140- 170 (150)	*	*

* Not observed.

Some specimens of nematodes obtained from hosts of the MPEG collection were post-fixed in 1% Osmium Tetroxide (OsO4), dehydrated, dried at the critical point of CO_2_ and coated in gold and analyzed under the Scanning Electron Microscope (SEM) LEO 1450VP from the Laboratory of Scanning Electron Microscopy of MPEG.

We had access to the type material of *P. retusa* from Rudolphi (1819) and the material from Ortlepp (1922). We analyzed two series of nematodes identified as *P. retusa*: one containing the type series described by Rudolphi (1819) (under the code NHM 6713), and other specimens obtained by Ortlepp (1922) (under the code NHM 17157), both deposited in the helminthological collection of Natural History Museum Vienna, Austria (Naturhistorisches Museum Wien - NHMW).

We also examined the following specimens from different helminthological collections under light microscopy for morphological comparisons: I. The French National Museum of Natural History (Muséum National D'Histoire Naturelle/France - MNHN) - *P. retusa* (MNHNIN-BA 166, 684, 686-689, 691-715, 985; MNHN-IN-ES-234, 309-2, MNHN-IN-F-1113, MNHN-IN-NJ-221, MNHN-IN-PK-29160); II. NHMW (Vienna/Austria) - *Physaloptera monodens* Molin, 1860 (NHM 6634, 6838) and *Physaloptera obtusissima* Molin, 1860 (NHM 6643); III. Natural History Museum of London, United Kingdom (BMNH) - *Physaloptera bonnei* Ortlepp, 1922 (BMNH 1998.11.26.68-70); IV. Helminthological Collection of the Oswaldo Cruz Institute, Brazil (CHIOC) - *Physaloptera bainae* Pereira, Alves, Rocha, Souza Lima & Luque, 2014 (CHIOC 35885b- d), *Physaloptera liophis* Vicente & Santos, 1974 (CHIOC 31034b-c, 31250b-j, 35801), *Physaloptera lutzi* Cristófaro, Guimarães & Rodrigues, 1976 (CHIOC 11488, 11111, 19244, 20606, 20980, 34836, 34837, 35111), *P. retusa* (CHIOC 34121a-c, 34124, 34142, 34838, 34679, 34680), and *Physaloptera tupinambae* Pereira, Alves, Rocha, Souza Lima & Luque, 2012 (CHIOC 35811b).

## Results

### *Physaloptera retusa* (Rudolphi, 1819) (Figures 1, 2, 3 and 4)

**Figure 1:**
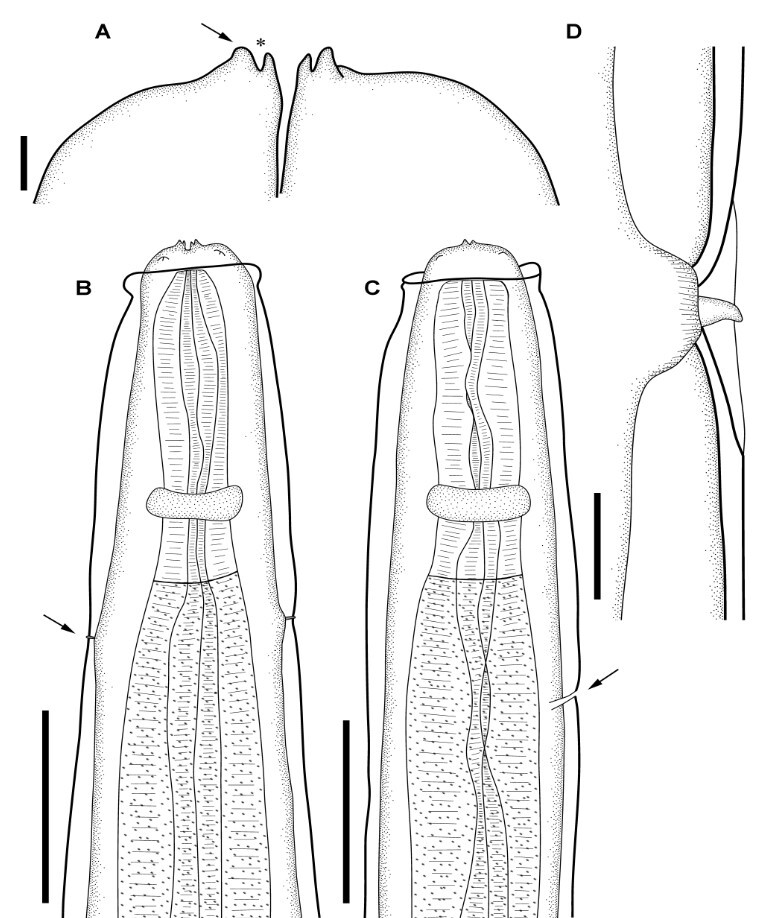
Line drawings of the type material of *Physaloptera retusa* (Rudolphi, 1819) by light microscopy. **(A)** Anterior extremity in lateral view, detailing the outer (*arrow*) and inner tooth (*asterisk*); **(B)** Anterior extremity of male, demonstrating the nerve ring and deirids (*arrow*) positions; **(C)** Anterior extremity of female, demonstrating the nerve ring and excretory pore (*arrow*) positions; **(D)** Detail of a deirid. *Scale-bars*: A: 10 µm; B, C: 200 µm; D: 20 µm.

**Figure 2:**
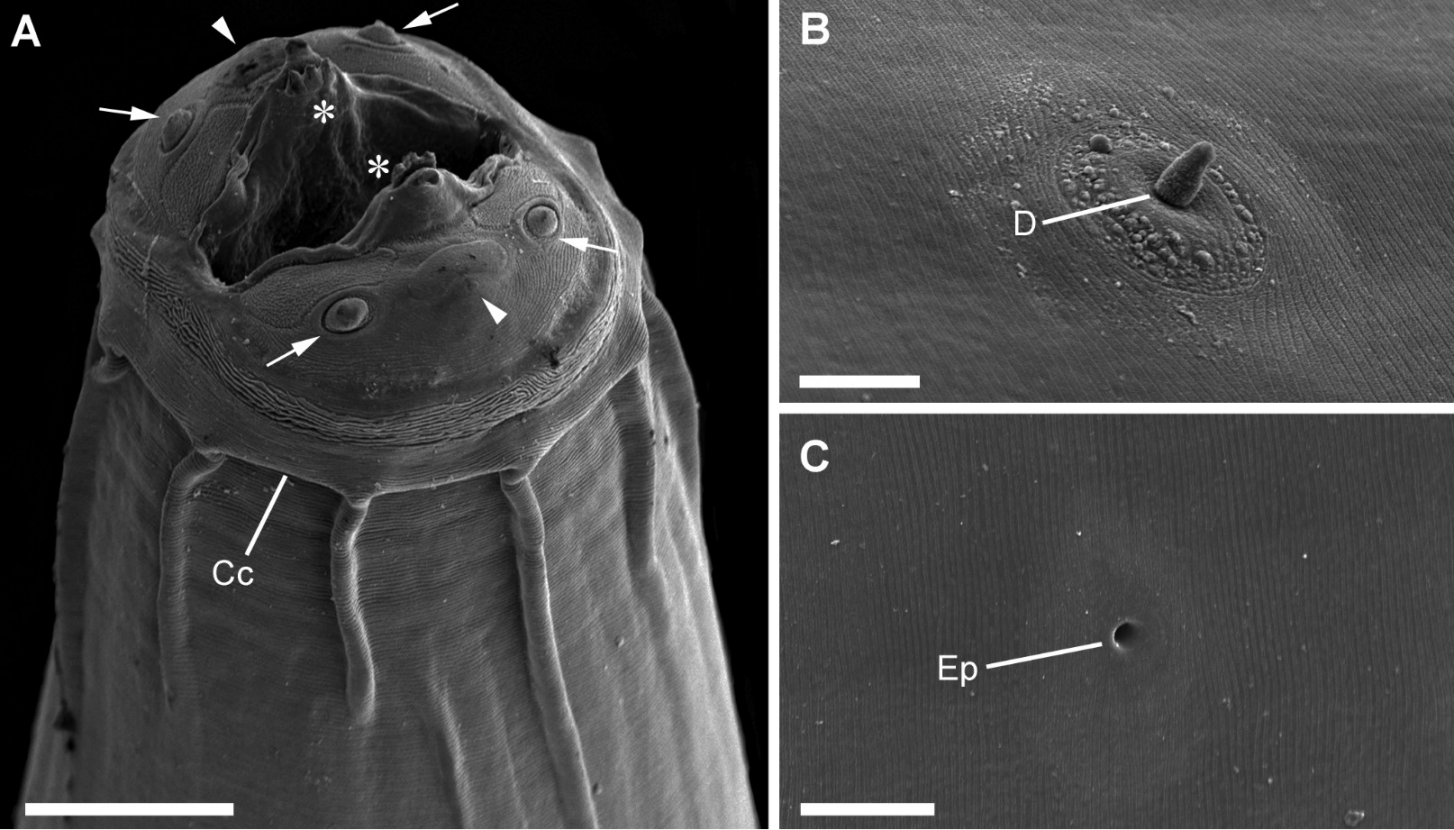
Scanning electron microscopy of *Physaloptera retusa.* **(A)** Anterior extremity; detailing the cephalic papillae (arrow), the amphids (arrowheads), the cephalic collar (Cc) and the tooth (asterisks); **(B)** Detail of a deirid (D); **(C)** Detail of the excretory pore (Ep). *Scale-bars*: A: 40 µm; B, C: 20 µm.

**Figure 3:**
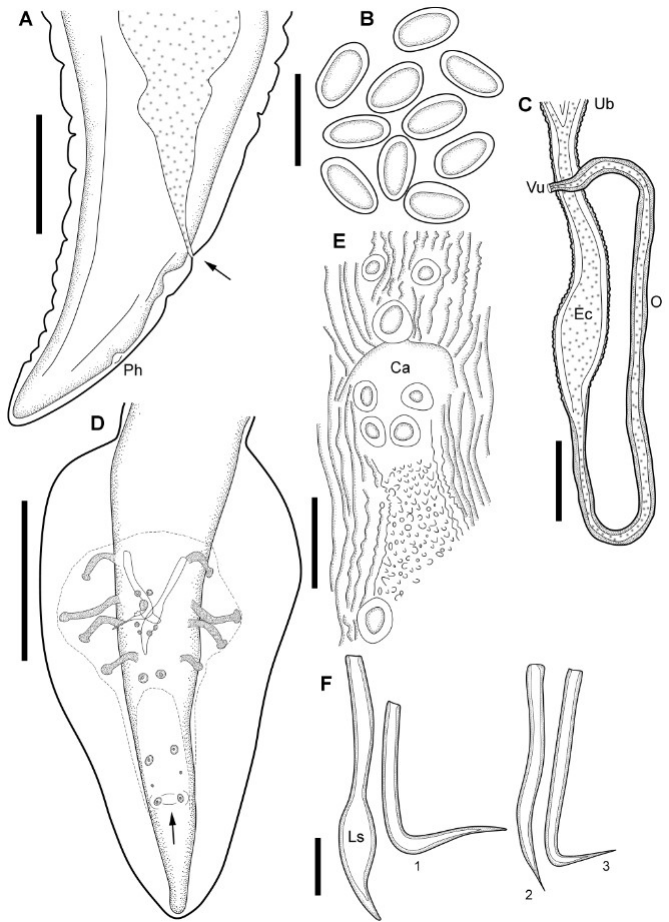
Line drawings of the type material of *Physaloptera retusa* (Rudolphi, 1819) by light microscopy*.* **(A)** Posterior extremity of female, showing the anus (arrow) and the phasmidial pore (Ph); **(B)** Eggs; **(C)** Genital tract of the female; demonstrating the common trunk, the egg chamber (Ec), the ovojector (O); the uterine branches (Ub) and the vulva (Vu); **(D)** Posterior extremity of male; showing the bursa and the caudal papillae and the “boss” between the last pair of sessile papillae (arrow); **(E)** Detail of cloacal aperture (Ca) , showing the surrounding papillae and the rough pattern of the cuticle; **(F)** Details of the spicules, spear shaped left spicule (Ls) and the right spicule in three different positions (1, 2 and 3). *Scale-bars*: A, C: 200 µm; B: 50 µm; E, F: 100 µm; D: 500 µm.

**Figure 4:**
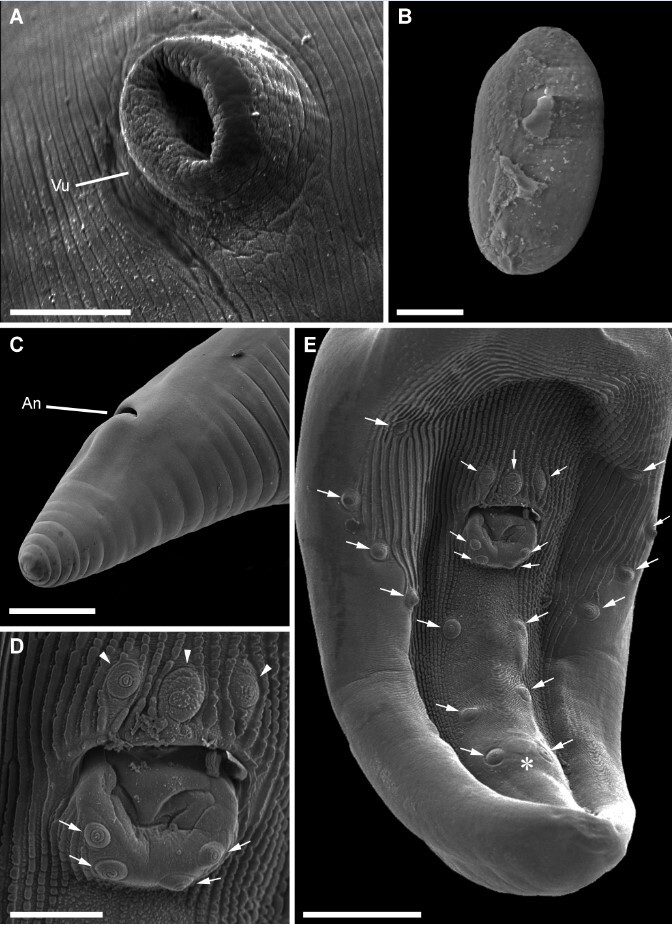
Scanning electron microscopy of *Physaloptera retusa.* **(A)** Detail of the vulva (Vu) opening; **(B)** Detail of an egg; **(C)** Posterior extremity of the female, showing the anus (An); **(D)** Details of the cloacal aperture and surrounding papillae (arrowhead); **(E)** Posterior extremity of the male, showing the cloaca and caudal papillae (arrow) and the “boss” (asterisk);. *Scale-bars*: A, C: 150 µm; B, D: 50 µm; E: 10 µm.

#### Redescription

General: Mouth surrounded by two symmetrical pseudolips (Figure 1A, 2A), each ornamented with a pair of submedian papillae and one amphid situated between the cephalic papillae (Figure 2A), Anterior extremity with an evident cephalic collar, forming a dilatation at anterior extremity, reflected over base of lips (Figures 1B, C, 2A). Internally to each pseudolip, one externo-lateral tooth and a tripartite interno-lateral tooth present (Figure 2A). Esophagus divided into a short muscular anterior portion and a long glandular posterior part (Figures 1B, C). Nerve-ring encircling muscular portion of esophagus (Figures 1B, C); deirids symmetrical, situated just posterior to the muscular-glandular junction of esophagus (Figures 1B, D, 2B) and excretory pore slightly posterior to deirids (Figures 1C; 2C). Sexual dimorphism evident, with females larger than males in size (see Table 1). Females with genital tract divided into ovijector, followed by the egg chamber and a common trunk (Figure 3C) ending in a protuberant vulva (Figure 4A), with eggs embryonated (Figures 3B, 4B). Tail rounded, mucronated, ending in a small conical process; phasmidial pore present in tail extremity (Figures 3A, 4C). In males, well-developed caudal bursa present, with 21 caudal papillae including: 4 pairs of pedunculate papillae, 3 sessile precloacal papillae (2 small papillae and 1 large unpaired papilla above cloacal aperture), 2 pairs of sessile ad-cloacal papillae on the posterior cuticular extremity of the cloaca, 3 equidistant pairs of caudal papillae on center of tail, and a “cuticular boss” similar to a papilla between last pair of papillae (Figures 3D, E, 4D, E). Phasmids paired situated between last two last pairs of sessile papillae (Figure 3D). Spicules subequal; right spicule slightly bent, ending in a thin tip and left spicule spear shaped (Figure 3F).

Table 1 presents all morphological data obtained from examining the type material and the material used for the first redescription of *P. retusa*, in addition, we also include new data from material collected in this study and data from the most recent redescription of this species.

#### Taxonomic summary

**Type-host:***Tupinambis teguixin* (Linnaeus, 1758) (Family Teiidae)

**Infection site:** Stomach

**Type-Locality:** Cuiabá, Mato Grosso, Brazil

**Examined material:** 5 males and 5 females of *P. retusa* from the Vienna Museum of Natural History, collected by Natterer in 1824 (nº4497) (code: NHM 6713). Voucher specimens of *P. retusa* were observed from Vienna Museum of Natural History (code: NHM 17157), Oswaldo Cruz Institute (codes: CHIOC 34121a-c, 34124, 34142, 34838, 34679 and 34680) and the French Museum of Natural History (codes: MNHN-IN-BA 166, 684, 686-689, 691-715, 985; MNHN-IN-ES-234, 309-2, MNHN-IN-F-1113, MNHN-IN-NJ-221, MNHN-IN-PK_29160). Additionally, we obtained 28 specimens of *P. retusa* from 15 specimens of *A. ameiva* from the herpetological collection of Emílio Goeldi Museum (MPEG) from Flona Caxiuanã, Melgaço, Pará, Brazil.

**Host records:***Ameiva ameiva* (Teiidae) (Poinar & Vaucher, 1972; Ribas et al., 1998; Ávila & Silva, 2010), *Ameivula nativo* Rocha, Bergallo & Peccinini-Seale (Teiidae) (Menezes et al., 2004), *Ameivula ocellifera* (Spix) (Teiidae) (Ávila & Silva, 2010), *Amphisbaena alba* Linnaeus (Amphisbaenidae) (Molin, 1860a), *Anolis fuscoauratus* D’Orbigny (Dactyloidae) (Goldberg et al., 2006; Ávila & Silva, 2010; Albuquerque et al., 2012), *Anolis trachyderma* Cope (Dactyloidae) (Albuquerque et al., 2012), *Aspronema dorsivittatum* (Cope) (Scincidae) (Rocha & Vrcibradic, 2003), *Brasiliscincus agilis* (Raddi) (Scincidae) (Ribas et al., 1998), *Chatogekko amazonicus* (Andersson) (Sphaerodactylidae) (Ávila & Silva, 2010), *Cnemidophorus lemniscatus* (Linnaeus) (Teiidae) (Ávila & Silva, 2010), *Copeoglossum nigropunctatum* (Spix) (Scincidae) (Ávila & Silva, 2010), *Enyalius bilineatus* (Duméril & Bibron) (Leiosauridae) (Vrcibradic et al., 2007), *Glaucomastix abaetensis* (Reis Dias, Rocha & Vrcibradic) (Teiidae) (Dias et al., 2005), *Glaucomastix littoralis* (Rocha, Bamberg Araújo & Vrcibradic) (Teiidae) (Ribas et al., 1995; Van Sluys et al., 2000; Dias et al., 2005; Ávila & Silva, 2010), *Gonatodes humeralis* (Guichenot) (Sphaerodactylidae) (Ávila & Silva, 2010), *Holcosus festivus* (Lichtenstein & Von Martens) (Teiidae) (Ávila & Silva, 2010), *Kentropyx altamazonica* (Cope) (Teiidae) (Ávila & Silva, 2010), *Kentropyx calcarata* Spix (Teiidae) (Goldberg et al., 2007; Ávila & Silva, 2009, 2010), *Kentropyx pelviceps* (Cope) (Teiidae) (Ávila & Silva, 2010; Albuquerque et al., 2012), *Liolaemus lutzae* Mertens (Liolaemidae) (Rocha, 1995), *Ophiodes striatus* (Spix) (Diploglossidae) (Molin, 1860a; Baker, 1987; Ávila et al., 2011), *Plica* (Linnaeus) (Tropiduridae) (Ávila & Silva, 2010), *Plica umbra* (Linnaeus) (Tropiduridae) (Ávila & Silva, 2010; Ávila & Silva, 2011; Albuquerque et al., 2012), *Potamites ecpleopus* (Cope) (Gymnophthalmidae) (Goldberg et al., 2007), *Salvator rufescens* (Günther) (Teiidae) (Ávila & Silva, 2010), *Tropidurus hispidus* (Spix) (Tropiduridae) (Prieto, 1980; Ávila et al., 2012), *Tupinambis longilineus* Ávila-Pires (Teiidae) (Ávila & Silva, 2010), *Tropidurus oreadicus* Rodrigues (Tropiduridae) (Ávila et al., 2011), *Tropidurus torquatus* Wied-Neuwied (Tropiduridae) (Vicente et al., 1993; Ribas et al., 1998; Van Sluys et al., 2000; Alves et al., 2022), *Tupinambis teguixin* (Teiidae) (Rudolphi, 1819; Diesing, 1851; Molin, 1860a; Ortlepp, 1922; Yamaguti, 1961; Ávila & Silva, 2010) and *Varzea bistriata* (Spix) (Scincidae) (Molin, 1860a).

#### Remarks

Ortlepp (1922) redescribed *P. retusa* using both type material and specimens obtained from a *T. teguixin* found dead in the gardens of the London Zoological Society. However, Ortlepp's redescription does not entirely correspond to what we observed analyzing individual specimens in the type series. The main differences between the specimens that were studied and deposited in the collections by Rudolphi and Ortlepp are the lengths of the spicules (420-470 right and 410-490 left in Rudolphi’s specimens *vs* 230-280 right and 265-310 left in Ortlepp’s specimens). In addition, the distribution pattern of cloacal papillae differ between the groups of specimens and from the center of the tail there are 3 equidistant pairs in Rudolphi’s specimen’s *vs* 3 asymmetrical pairs in Ortlepp’s specimens. Finally, the limits of the ornamentation of the caudal bursa is different in extent measuring 2.0-2.3 × 1.1-1.4 mm in Rudolphi’s specimens *vs* 1.6-2.0 × 0.8-1.2 mm in Ortlepp’s collection.

The right spicule of males is not "hooked," as indicated by Ortlepp. We observed that it may assume rectilinear or more curved shapes according to the position of the specimens on the slides (Figure 3F). The morphological trait "straight hooked spicule" does not seem to be a character that can be used to discriminate male specimens of *P. retusa* from other species. Also, Ortlepp reported a relatively short ovijector for female specimens of *P. retusa* compared to our observations of 1.4-1.8 mm for Rudolphi’s *vs.* 0.62-0.9 mm for Ortlepp’s. In addition, the vulva ratios are quite different between the two specimen groups, namely 16% in Rudolphi’s specimens *vs.* 23% in those studied by Ortlepp. Thus, our measurements better correspond to the original specimens from the type-series.

The redescription of *P. retusa* by Alves et al. (2022) agrees with the description by Rudolphi (1819) regarding the spicules' morphology and the caudal papillae distribution pattern. However, the male specimens described by Alves et al. (2022) are smaller than our observed measurements of the type material in the following: body length (18-38 mm Rudolphi *vs.* 13.5-16.1 mm Alves, Couto & Pereira), maximum width (650-800 Rudolphi *vs.* 377-650 Alves, Couto & Pereira), length of the esophagus (3.6-5.9 mm Rudolphi *vs*. 2.6-2.9 mm Alves, Couto & Pereira), tail (1.2-1.4 mm Rudolphi *vs*. 0.547-0.800 mm Alves) and spicules (420-470 right and 410-490 left Rudolphi *vs.* 277-344 right and 287-380 left Alves, Couto & Pereira). Additionally, both the morphological and morphometric data of the specimens collected in this study from *Ameiva ameiva* agree with our observations of the type material of *P. retusa*.

Among the didelphic species group of the genus *Physaloptera*, 19 species were reported from Neotropical hosts and only 9 are parasitic in reptiles, namely: *P. bainae*; *P. bonnie*; *P. liophis*; *P. lutzi*; *P. monodens*; *P. nordestina* Matias, Morais & Ávila, 2020; *P. obtusissima*; *P. retusa* and *P. tupinambae*. Therefore, we compared our observations of type material of *P. retusa* only with the other parasitic species occurring in neotropical reptiles.

In comparison to *P. retusa*, *P. lutzi* is the most different regarding the morphology of oral structures (with a variable number of spikes in both inner and outer teeth), the position of the vulva (on the posterior third of the body, corresponding to 95% of the body length) and the length of spicules (the right spicule has half the length of the left, with a ratio of 1:1.8-2.0). Recently Alves et al. (2022) redescribed *P. lutzi* from *T. torquatus* and our observations of this species are congruent with these authors.

*Physaloptera retusa* also differs from *P. bonnei* and *P. liophis* by the vulva position (both subequatorial, 40% and 54.2% of body length respectively *vs.* 16% in *P. retusa*) and from *P. liophis* by the length of spicules (420-470 right and 410-490 left in *P. retusa vs.* 250 right and 260 left in *P. liophis*).

*Physaloptera retusa* can be easily differentiated from *P. bainae* and *P. tupinambae* when comparing the number of male caudal papillae; these are the only two species parasitic in neotropical reptiles with more than 21 papillae (23 and 22 respectively *vs.* 21 in *P. retusa*). Based on the morphology of oral structures, *P. bainae* is easily differentiated by having an outer tooth with four small spines in a cross-shaped pattern; and *P. tupinambae* differs by the presence of a bipartite internal tooth, while in *P. retusa* the outer tooth is triangular, and the inner tooth is tripartite.

*Physaloptera retusa* can be differentiated from *P. monodens* and *P. obtusissima* by the spicules' length and the oral structures' morphology. The spicules are larger in *P. retusa* (420-470 right and 410-490 left) than in *P. monodens* (362 right and 415 left) and *P. obtusissima* (385 right and 430 left); and the outer tooth in these species is conical, while that in *P. retusa* is triangular in shape.

*Physaloptera retusa* differs from *P. nordestina* by the shape of the outer tooth (triangular in *P. retusa vs.* conical in *P nordestina*), length of the spicules (420-470 right and 410-490 left in *P. retusa vs.* 195-376 right and 257-436 left in *P. nordestina*) and vulva ratio (16% of the body length in *P. retusa vs.* 5-26% of the body length in *P. nordestina*).

*Physaloptera mucronata* Leidy, 1856 was described based on specimens collected from *Melanosuchus niger* (Spix) (Alligatoridae) in Brazil (Diesing, 1851). The species was reported in *Alligator mississippiensis* (Daudin) (Alligatoridae) from the United States by Leidy (1856), which was considered synonymous with *P. retusa* (Walton, 1927). However, the species was renamed to *Ascaris lanceolata* by Molin (1860b) and posteriorly redescribed. Subsequently, the species was assigned to the genus *Terranova* by Sprent (1979) and, most recently, reassigned to *Neoterranova lanceolata* (Molin) by Moravec & Justine (2020). Thus, we did not compare this species with *P. retusa*.

Pereira et al. (2012) suggested that the determination of the number of caudal papillae in the males of *P. retusa* in the study of Vicente et al. (1993) were quite different compared to the original description of Rudolphi (1819) and the subsequent studies of Ortlepp (1922) and Skrjabin & Sobolev (1964), which may have led and may in the future, lead to additional misidentifications. These references are essential keys for the taxonomic identification of *P. retusa*. Thus, researchers using these references for species identification should be careful.

We also observed differences regarding the morphology of *P. liophis*. This species is closely related to *P. retusa* but differ mainly by the number of caudal papillae (23 papillae in total, 8 pedunculate, and 15 sessile in *P. liophis vs.* 21 papillae in total, 8 pedunculate and 13 sessile in *P. retusa*). However, we did not observe these extra papillae in the type material of *P. liophis*, and according to the illustrations provided in the original description of *P. liophis*, we hypothesized that the authors probably included the phasmidial pores along with caudal papillae. Thus, we consider that *P. liophis* has 21 papillae (8 pedunculate and 13 sessile) instead of the 23 papillae previously indicated (Pereira et al., 2012, 2014). Also, the inner tooth morphology, not mentioned in the original description, remains unknown, and we could not observe it because of the poor preservation quality of the specimens.

Additional morphological and morphometric data of *Physaloptera* spp. parasites of reptiles from Neotropics are presented in Table 2.

**Table 2:** Comparison of the main morphological and morphometric characters used in the identification of *Physaloptera* spp.

Species	Locality	Outer tooth morphology	Inner tooth morphology	Vulva position	Vulva ratio (%)	Left spicule	Right spicule	Number of papillae	Reference
*P. retusa*	Brazil	Large, triangular	Large, tripartite	Anterior third	16	475	455	21 (8 st, 13 se)	This study, Ortlepp (1922)
*P. bainae*	Brazil	Four spines	Medium, tripartite	Anterior third	20.2-22.6	589-617	569-600	23 (8 st, 15 se)	Pereira et al. (2014)
*P. bonnei*	Suriname	Conical obtuse	Small, tripartite	Half third	40	455	455	21 (8 st, 13 se)	Ortlepp (1922)
*P. liophis*	Brazil	Large, triangular	Not reported	Half third	54.2	260	250	21 (8 st, 13 se)	Vicente & Santos (1974),
*P. lutzi*	Brazil	Variable number of spikes	Variable number of spikes	Posterior third	94.0-94.7	460-560	250-280	21 (8 st, 13 se)	Guimarães et al (1976)
*P. monodens*	Brazil	Small, conical	Small, tripartite	Anterior third	20	415	362	21 (8 st, 13 se)	Molin (1860a), Ortlepp (1922)
*P. nordestina*	Brazil	Large, conical	Small, tripartite	Anterior third	15-26	257-436	195-376	21 (8 st, 13 se)	Matias et al. (2020)
*P. obtusissima*	Brazil	Large, conical	Large, tripartite	Anterior third	25	430	385	21 (8 st, 13 se)	Molin (1860a), Ortlepp (1922)
*P. tupinambae*	Brazil	Medium, rounded	Medium, bipartite	Anterior third	25.0-26.3	558-585	528-540	22 (8 st, 14 se)	Pereira et al. (2012)

st = stalked papillae, se = sessile papillae.

## Discussion

The presence of a cephalic collar at the anterior extremity, two lateral pseudolips, with an external tooth and an internal tripartite tooth, and the pattern of papillae of the male caudal region, namely, a caudal bursa ornamented with 21 caudal papillae are the main characters of the genus *Physaloptera* (Rudolphi, 1819; Ortlepp, 1922; Skrjabin & Sobolev, 1964; Chabaud, 2009). This genus includes more than 100 species widely distributed globally, of which several remain insufficiently described, hampering comparisons and species differentiation (Pereira et al., 2012, 2014).

*Physaloptera* spp. females have a variable number of uterine branches. Ortlepp (1922) highlighted that this might be an essential character for species identification. Thus, several authors separated these species into groups according to the type of uteri: didelphic (two branches); tridelphic (three branches), or tetradelphic (four branches) (Ortlepp,1922; Ortlepp, 1937; Morgan, 1943; Skrjabin & Sobolev, 1964; Chabaud, 2009). In the Neotropical region, species of *Physaloptera* parasitic in reptiles typically have only two uterine branches. Thus, we compared our specimens with the 9 species of the didelphic group of these nematodes parasitic in Neotropical reptiles.

Our scanning electron microscopy (SEM) analysis revealed ultrastructural details of important characters for species diagnosis. Using this method, we confirmed the details of both the inner and outer tooth morphology and the distribution of caudal papillae of *P. retusa*. The use of SEM as a tool for helminth taxonomy has been helping for a better comprehension of the morphology of several *Physaloptera* spp. (Marchiondo & Sawyer , 1978; Tiekotter, 1981; Mafra & Lanfredi, 1998; Lopes-Torres et al., 2009; Naem & Asadi, 2013; Chen et al., 2017; Ederli et al., 2018; Lopes-Torres et al., 2019, Maldonado et al., 2019; Matias et al., 2020). But, studies of physalopterid nematodes using both light microscopy and SEM are scarce (Naem & Asadi, 2013) and we reinforce the fact that further studies of other physalopterid species using SEM may help to define and solidify the real taxonomic value of other characters as suggested by Lopes-Torres et al. (2009).

The naturalist Johann Natterer collected specimens of *P. retusa* from Cuiabá, Brazil, and later this material was sent to NHMW, and Rudolphi formally described the species in 1819 (see historical summary in Guerrero, 2021). However, the author did not give morphological details for *P. retusa*, and the species remained insufficiently described until the work of Ortlepp (1922). Our analysis on the type material of this species are congruent with the redescription of Ortlepp (1922) regarding the dimensions of males, the length of spicules, the distribution and pattern of male cloacal papillae, the morphology of the cloacal aperture, and the limits of the caudal bursa.

We observed differences in the morphology of the right spicule (this spicule is maleable, thin and weakly esclerotized, thus its morphology may varies according to the position of the nematode on the slide), as well as the length of the ovijector in females and other morphometric data. These differences might be associated with the limitation of bidimensional analyzes of internal structures with a complex morphological organization and/or intraspecific morphological variation. Lopes-Torres et al., (2019) conducted an integrative study presenting three-dimensional information of morphological characters of *Physaloptera mirandai* Lent & Freitas, 1937. Despite the fact that we did not use three-dimensional techniques, our study includes new morphometric and morphological details of important characters obtained from recently collected specimens and type series that can be used to identify and differentiate *P. retusa* from its congeners. The combination of multidimensional techniques may help to better comprehend the morphological complexity of important taxonomic characters.

The differences observed between specimens of different hosts (*T. teguxin* and *A. ameiva*) might be related to different factors related to hosts, the helminths, and their host-parasite relationships. Some specimens or even species could have more equilibrated host-parasite relationships compared to the others and affect the parasite development. Chitwood (1957) and Haley (1962) listed some factors that may cause intraspecific variations in helminths’ morphological and morphometric characters, such as host age, diet, metabolic and physical condition, number of parasites, presence of other parasite species, etc. Also, geographic, and ecological factors could influence species development and cause intraspecific variations (Chitwood, 1957). Some of these factors are difficult to evaluate and remain unstudied for several groups of parasites. Regarding nematode parasites, there are a small number of studies pointing and discussing their morphological variability in different host species and localities exist. Therefore, we think that differences in the physiology and ecology of the hosts and the phenotypic plasticity of the nematode might explain the variations observed.

*Physaloptera retusa* is the most common species of the genus in neotropical lizards, occurring in several host families (Ávila & Silva, 2011; Ávila et al., 2011, 2012; Albuquerque et al., 2012). Therefore, all morphological and morphometrical variation observed suggest that *P. retusa* represents a set of cryptic species (or even a species complex) that still needs to be revised using the integrative taxonomy, exploring a combination of molecular and morphological studies.

Pereira et al. (2012) described *P. tupinambae* with 22 papillae in total, resulting from an extra unpaired papilla in place of the “boss” between the last two sessile papillae. Drawings and photomicrographs provided by the authors indicate the presence of this character. The “boss” located between the last pair of sessile papillae seems to be a typical morphological character for some species, and the males of *P. liophis*, *P. monodens* and *P. retusa* have this structure. Nevertheless, it was not possible to identify an extra papilla in the descriptions of these species, which is congruent with our observations of the papillae distribution and ultrastructural details of the “boss” of *P. retusa* under SEM. Additionally, studies on *Physaloptera* spp. from mammals also observed the “boss” in the male caudal region, and this extra unpaired papilla is not present (Norman & Beveridge, 1999). Thus, *P. bainae* and *P. tupinambae* are the only species parasitic in reptiles with a different number of caudal papillae. However, additional morphological studies are necessary to confirm this character since it can be easily confused with the rough pattern present in the tail and cloacal aperture of these nematodes.

There are many problems in character definition relative to species delimitation, which may lead to error cascades as also pointed out by Bortolus (2008). Thus, once an error in measurement or interpretation enters the literature stream without correction, inaccurate information may be propagated down through time throughout the literature, which is particularly frequent in parasitology (see Van Bortel et al., 2001; Vink et al., 2012).

Use of archived museum specimens for reexamination of previously described taxa with more detailed descriptions and additional data are still necessary and may help solve taxonomic problems, increasing our knowledge of biodiversity and will improve and establish more accurate species identifications. Therefore, the problems within *Physaloptera* presented herein demonstrate the necessity of additional studies of both museum collections and new collections in the field that will serve to elucidate species diversity in the genus. Also, integrative approaches combining different sources of information and complementary perspectives are necessary to improve our comprehension of the morphological complexity of *Physaloptera* species and to improve helminth systematics.

## References

[B001] Albuquerque S, Ávila RW, Bernarde PS (2012). Occurrence of helminths in lizards (Reptilia: Squamata) at Lower Moa River Forest, Cruzeiro do Sul, Acre, Brazil. Comp Parasitol.

[B002] Alves PV, Couto JV, Pereira FB (2022). Redescription of two most recorded *Physaloptera* (Nematoda: Physalopteridae) parasitizing lizards in the Americas: first step towards a robust species identification framework. Syst Parasitol.

[B003] Ávila RW, Anjos LA, Ribeiro SC, Morais DH, Silva RJ, Almeida WO (2012). Nematodes of lizards (Reptilia: Squamata) from Caatinga Biome, northeastern Brazil. Comp Parasitol.

[B004] Ávila RW, Cardoso MW, Oda FH, Silva RJ (2011). Helminths from lizards (Reptilia: Squamata) at the Cerrado of Goiás state, Brazil. Comp Parasitol.

[B005] Ávila RW, Silva RJ (2010). Checklist of helminths from lizards and amphisbaenians (Reptilia, Squamata) of South America. J Venom Anim Toxins Incl Trop Dis.

[B006] Ávila RW, Silva RJ (2011). Helminths of lizards (Reptilia: Squamata) from Mato Grosso State, Brazil. Comp Parasitol.

[B007] Ávila RW, Silva RJ (2009). Helminths of the teiid lizard *Kentropyx calcarata* (Squamata) from an Amazonian site in western Brazil. J Helminthol.

[B008] Baker MR (1987). Synopsis of the Nematoda parasitic in amphibians and reptiles.

[B009] Bortolus A (2008). Error cascades in the biological sciences: the unwanted consequences of using bad taxonomy in ecology. Ambio.

[B010] Chabaud AG, Anderson RC, Chabaud AG, Wilmott S (2009). Keys to the nematode parasites of vertebrates: archival volumes..

[B011] Chen HX, Ju HD, Li Y, Li L (2017). Further study on *Physaloptera clausa* Rudolphi, 1819 (Spirurida: Physalopteridae) from the Amur hedgehog *Erinaceus amurensis* Schrenk (Eulipotyphla: Erinaceidae). Acta Parasitol.

[B012] Chitwood MB (1957). Intraspecific variation in parasitic nematodes. Syst Zool.

[B013] Davis E, Beane JC, Flowers JR (2016). Helminth parasites of pit vipers from North Carolina. Southeast Nat.

[B014] Dias EJR, Vrcibradic D, Rocha CFD (2005). Endoparistes infecting two species of whiptail lizard (*Cnemidophorus abaetensis* and *C. ocellifer*; Teiidae) in a ‘Restinga’ habitat of North-eastern Brazil. Herpetol J.

[B015] Diesing CM (1851). Systerna Helminthum.

[B016] Ederli NB, Gallo SSM, Oliveira LC, Oliveira FCR (2018). Description of a new species *Physaloptera goytaca* n. sp. (Nematoda, Physalopteridae) from *Cerradomys goytaca* Tavares, Pessôa & Gonçalves, 2011 (Rodentia, Cricetidae) from Brazil. Parasitol Res.

[B017] Esteban JG, Botella P, Toledo R (1995). Redescription of *Physaloptera brevivaginata* Seurat, 1917 (Nematoda: Physalopteridae) from the bat *Myotis blynthii* (Tomes) (Chiroptera: Vespertilionidae) in Spain. Syst Parasitol.

[B018] Gardner SL, Fisher RN, Barry SJ (2012). Collecting and preserving parasites during reptile biodiversity surveys..

[B019] Goldberg SR, Bursey CR, Caldwell JP, Vitt LJ, Costa GC (2007). Gastrointestinal helminths from six species of frogs and three species of lizards, sympatric in Pará state, Brazil. Comp Parasitol.

[B020] Goldberg SR, Bursey CR, Vitt LJ (2006). Helminths of the Brown-eared anole, *Norops fuscoauratus* (Squamata, Polychrotidae), from Brazil and Ecuador, South America. Phyllomedusa.

[B021] Guerrero R (2021). Natterer in neotropical nematoda: species described by Rudolphi, Diesing, and Molin. MANTER: Journal of Parasite Biodiversity.

[B022] Guimarães JF, Cristofaro R, Rodrigues HO (1976). Alguns nematódeos de *Tropidurus torquatus* e *Ameiva ameiva* - Fauna helmintológica de Salvador, Bahia. Atas Soc Biol.

[B023] Haley AJ (1962). Role of host relationships in the systematics of helminth parasites. J Parasitol.

[B024] Leidy J. (1856). A synopsis of entozoa and some of their ecto-congeners observed by the author.

[B025] Lopes-Torres EJ, Girard-Dias W, Mello WN, Simões RO, Pinto IS, Maldonado A (2019). Taxonomy of *Physaloptera mirandai* (Nematoda: Physalopteroidea) based in three-dimensional microscopy and phylogenetic positioning. Acta Trop.

[B026] Lopes-Torres EJ, Maldonado A, Lanfredi RM (2009). Spirurids from *Gracilianus agilis* (Marsupialia: Didelphidae) in Brazilian Pantanal wetlands with a new species of *Physaloptera* (Nematoda: Spirurida). Vet Parasitol.

[B027] Mafra AC, Lanfredi RM (1998). Reevaluation of *Physaloptera bispiculata* (Nematoda: Spiruroidea) by light and scanning electron microscopy. J Parasitol.

[B028] Maldonado A, Simões RO, Luiz JS, Costa-Neto SF, Vilela RV (2019). A new species of *Physaloptera* (Nematoda: Spirurida) from *Proechimys gardneri* (Rodentia: Echimyidae) from the Amazon rainforest and molecular phylogenetic analyses of the genus. J Helminthol.

[B029] Marchiondo AA, Sawyer TW (1978). Scanning electron microscopy of head region of *Physaloptera felidis* Ackert, 1936. Proc Helminthol Soc Wash.

[B030] Matias CSL, Morais DH, Ávila RW (2020). *Physaloptera nordestina* n. sp. (Nematoda: Physalopteridae) parasitizing snakes from Northeastern Brazil. Zootaxa.

[B031] Menezes VA, Vrcibradic D, Vicente JJ, Dutra GF, Rocha CFD (2004). Helminths infecting the partenogenetic whiptail lizard *Cnemidophorus nativo* in a restinga habitat of Bahia State, Brazil. J Helminthol.

[B032] Molin R (1860). Una monografia del genere Physaloptera..

[B033] Molin R (1860). Trenta specie di Nematoidi. Sitzungsberichte der Kaiserlichen Akademis der Wissenschaften in Wien. Mathematisch-Naturwissenschaftliche Classe.

[B034] Moravec F, Justine JL (2020). Erection of *Euterranova* n. gen. and *Neoterranova* n. gen. (Nematoda, Anisakidae), with the description of *E. dentiduplicata* n. sp. and new records of two other anisakid nematodes from sharks off New Caledonia. Parasite.

[B035] Morgan BB (1943). The *Physaloptera* (Nematoda) of rodents. Wasmann Collector.

[B036] Naem S, Asadi R (2013). Ultrastructural characterization of male and female *Physaloptera rara* (Spirurida: Physalopteridae): feline stomach worms. Parasitol Res.

[B037] Norman RJB, Beveridge I (1999). Redescriptions of the species of *Physaloptera* Rudolphi, 1819 (Nematoda: Spirurida) parasitic in bandicoots (Marsupialia: Perameloidea) in Australia. Syst Parasitol.

[B038] Ortlepp R (1937). Some undescribed species of the nematode genus *Physaloptera* Rud., together with a key to the sufficiently known forms. Onderstepoort J Vet Res.

[B039] Ortlepp RJ (1922). The nematode genus *Physaloptera* Rud*. Proc Zool Soc Lond.

[B040] Pereira FB, Alves PV, Rocha BM, de Souza Lima S, Luque JL (2014). *Physaloptera bainae* n. sp. (Nematoda: Physalopteridae) Parasitic in *Salvator merianae* (Squamata: Teiidae), with a Key to *Physaloptera* Species Parasitizing Reptiles from Brazil. J Parasitol.

[B041] Pereira FB, Alves PV, Rocha BM, Souza Lima S, Luque JL (2012). A new *Physaloptera* (Nematoda: Physalopteridae) parasite of *Tupinambis merianae* (Squamata: Teiidae) from Southeastern Brazil. J Parasitol.

[B042] Poinar GO, Vaucher C (1972). Cycle larvaire de *Physaloptera retusa* Rudolphi, 1819 (Nematoda, Physalopteridae), parasite d’un lezard sud-americain. Bull Mus Natl Hist Nat.

[B043] Prieto AS (1980). Note on parasites of the tropical lizard *Tropidurus hispidus.*. J Herpetol.

[B044] Ribas SC, Rocha CFD, Teixeira-Filho PF, Vicente JJ (1995). Helminths (Nematoda) of the lizard *Cnemidophorus ocellifer* (Sauria: Teiidae): Assessing the effect of rainfall, body size and sex in the nematode infection rates. Cienc Cult.

[B045] Ribas SC, Rocha CFD, Teixeira-Filho PF, Vicente JJ (1998). Nematode infection in two sympatric lizards (*Tropidurus torquatus* and *Ameiva ameiva*) with different foraging tactics. Amphib-Reptil.

[B046] Rocha CFD, Vrcibradic D (2003). Nematode assemblage of some insular and continental lizard host of the genus *Mabuya* Fitzinger (Reptilia, Scincidae) along the eastern Brazilian coast. Rev Bras Zool.

[B047] Rocha CFD (1995). Nematode parasites of the Brazilian sand lizard, *Liolaemus lutzae.*. Amphib-Reptil.

[B048] Rudolphi KA (1819). Entozoorum synopsis cui accedunt mantissa duplex et indices locupletissimi..

[B049] São Luiz J, Simões RO, Torres EL, Barbosa HS, Santos JN, Giese EG (2015). A new species of *Physaloptera* (Nematoda: Physalopteridae) from *Cerradomys subflavus* (Rodentia: Sigmodontinae) in the Cerrado Biome, Brazil. Neotrop Helminthol.

[B050] Skrjabin KI, Sobolev AA (1964). Principles of nematology XII. Spirurates of animal and man and the diseases caused by them, II Physalopteroidea..

[B051] Sprent JFA (1979). Ascaridoid nematodes of amphibians and reptiles: *Terranova.*. J Helminthol.

[B052] Tiekotter KL (1981). Observation of the head and tail regions of male *Physaloptera praeputialis* von Linstow, 1889 and *Physaloptera rara* Hall and Wigdor, 1918, using scanning electron microscopy. Proc Helminthol Soc Wash.

[B053] Van Bortel W, Harbach RE, Trung HD, Roelants PA, Backeljau TH, Coosemans MA (2001). Confirmation of *Anopheles varuna* in Vietnam, previously misidentified and mistargeted as the malaria vector *Anopheles minimus.*. Am J Trop Med Hyg.

[B054] Van Sluys M, Hatano F, Vicente J, Galdino CAC, Cunha-Barros M, Vrcibradic D (2000). Nematode infection patterns in four sympatric lizards from a restinga habitat (Jurubatiba) in Rio de Janeiro state, southeastern Brazil. Amphib-Reptil.

[B055] Vicente JJ, Rodrigues HO, Gomes DC, Pinto RM (1993). Nematóides do Brasil. Parte III: nematóides de répteis. Rev Bras Zool.

[B056] Vicente JJ, Santos E (1974). Sobre um novo nematódeo do gênero *Physaloptera Rudolphi*, 1819 parasito de cobra d’água (Nematoda, Spiruroidea). Atas Soc Biol.

[B057] Vink CJ, Paquin P, Cruickshank RH (2012). Taxonomy and irreproducible biological science. Bioscience.

[B058] Vrcibradic D, Vicente JJ, Bursey CD (2007). Helminths infecting the lizard *Enyalius bilineatus* (Iguanidae: Leisosaurinae) from an Atlantic Rainforest area in Espírito Santo state, southeastern Brazil. Amphib-Reptil.

[B059] Yamaguti S (1961). Systema Helminthum, the nematodes of vertebrates..

